# Optimizing oral 3-hydroxybutyrate dosage using pharmacokinetic model to improve cognitive function and mood in healthy subjects

**DOI:** 10.3389/fnut.2024.1470331

**Published:** 2025-01-08

**Authors:** Kentaro Nishioka, Takahiro Ishimoto, Mariko Sato, Ruki Yasuda, Yumi Nakamura, Hiroshi Watanabe, Toshihide Suzuki, Yudai Araragi, Yukio Kato, Ken-ichi Yoshida, Norihito Murayama

**Affiliations:** ^1^Research Institute, Suntory Global Innovation Center Ltd., Kyoto, Japan; ^2^Faculty of Pharmacy, Institute of Medical, Pharmaceutical and Health Sciences, Kanazawa University, Kanazawa, Japan; ^3^Department of Science, Technology and Innovation, Kobe University, Kobe, Japan

**Keywords:** ketone, 3-hydroxybutyrate, beta-hydroxybutyrate, brain energy, cognitive function, mood, compartment model

## Abstract

**Introduction:**

The brain uses ketones, mainly 3-hydroxybutyrate (3-HB), as an alternative energy source. Therefore, oral intake of 3-HB may help maintain brain health. Previous studies indicated that achieving a maximum concentration (C_max_) of 3-HB in plasma at 0.28 mM could initiate ketone metabolism in the brain; we hypothesized that attaining this C_max_ would improve brain health.

**Methods:**

We aimed to demonstrate the efficacy of an optimized single oral dose of 3-HB on cognitive function and mood through two clinical studies: a pharmacokinetic study and an efficacy study. In the pharmacokinetic study, healthy subjects were ingested 2 and 4 g of 3-HB to construct a compartment model to predict the minimum oral dose of 3-HB needed to achieve the target C_max_. In the efficacy study, a randomized, double-blinded, and placebo-controlled crossover trial, the effects of 3-HB at the predicted doses on cognitive function and mood in healthy subjects were assessed by a serial arithmetic test (SAT), the cognitrax, the profile of mood states 2nd edition (POMS2), and fatigue visual analog scale (VAS).

**Results:**

In the pharmacokinetic study, a one-compartment model that includes saturable and non-saturable absorption pathways, constant biosynthesis, and the linear elimination of 3-HB after oral administration were constructed. The model principally reflected the observed serum 3-HB concentrations profiles and predicted a minimum dose of 3.5 g needed to achieve the target C_max_. In the efficacy study, although no significant difference was observed in any cognitive domains assessed by the Cognitrax, total responses and correct answers in the SAT were significantly improved in the active group receiving 3.5 g of 3-HB compared to the placebo group. Regarding the POMS2, confusion–bewilderment, fatigue–inertia, vigor-activity, and total mood disturbance scales were significantly improved in the active group compared to the placebo group. Additionally, fatigue VAS were also significantly improved in the active group compared to the placebo group.

**Discussion:**

We successfully established a one-compartment model for oral 3-HB intake and demonstrated partial efficacy on cognitive function and broad efficacy on mood in healthy subjects with a single oral dose of 3.5 g of 3-HB optimized by the model.

**Clinical trial registration:**

https://www.umin.ac.jp/ctr/index-j.htm, identifier [UMIN000042095, UMIN000046666].

## 1 Introduction

The human brain has high energy requirements and consumes 20–25% of the total energy of the human body while constituting only 2% of its mass (1). Glucose is the primary energy source for the brain whereas its utilization declines with age, which correlates to decreased cognitive function and mood, as well as the onset of dementia and depression (2, 3). Under specific conditions such as starvation, the brain uses ketones, mainly 3-hydroxybutyrate (3-HB), produced from fatty acids in the liver, as an energy source (4). Ketone utilization is less affected by aging than glucose (5), prompting interest in strategies that supply ketones to the brain to support its health (6, 7).

There are two main methods of ketone administration through oral ingestion: adopting a ketogenic diet or consuming exogenous ketones (8). The ketogenic diet, which is low in carbohydrates and high in fats, promotes the production of ketones in the liver and has demonstrated efficacy in treating brain disorders, including Alzheimer’s disease (9). However, its strict dietary requirements can make it challenging to maintain ketone levels with this method (10). As a more manageable alternative, the intake of exogenous ketones, such as medium-chain triglycerides (MCT), 3-HB esters, and 3-HB itself, has attracted considerable attention (11). Among them, oral intake of 3-HB is generally considered the most physiologic way to increase ketone levels in the body. With reference to the available literature, only two studies have evaluated the effects of orally administered 3-HB on the brain in humans. Both reports demonstrated that 3-HB intake had no significant efficacy on cognitive function in healthy subjects (12, 13). However, these studies evaluated the effects of 3-HB under the particular circumstances of ergometer exercise loading conditions; therefore, the independent effects of 3-HB on cognitive function may be unclear. Next, regarding the dosage of 3-HB, the efficacy is likely to increase with increasing doses whereas smaller doses are more effective at mitigating side effects and flavor issues. Previous findings by Xin et al. revealed that 10 g of MCT could induce brain ketone metabolism with a maximum concentration (C_max_) of 3HB in plasma at 0.28 mM (14). Therefore, the minimum dosage of 3-HB that can achieve this C_max_ may possibly improve brain health with little risk of side effects and flavor issues. However, the pharmacokinetics of orally administered 3-HB have not yet been studied.

In this research, we aimed to evaluate the effects of an optimized oral single dose of 3-HB on cognitive function and mood through two clinical studies: a pharmacokinetic study and an efficacy study. In the pharmacokinetic study, healthy subjects were ingested 2 and 4 g of 3-HB to construct a compartment model to predict the minimum oral dose of 3-HB needed to achieve the target C_max_ of 0.28 mM. Given that 3.5 g of 3-HB was estimated to be the effective dosage, in the efficacy study, a randomized, double-blinded, and placebo-controlled crossover trial, we evaluated the effects of 3.5 g of 3-HB on cognitive function and mood in middle-aged healthy subjects, who may experience a decline in glucose metabolism during the early stages (15). For the assessment of cognitive function, we used serial arithmetic test (SAT) using the Uchida–Kraepelin test forms, which primarily measures attentional function, and the Congnitrax, a comprehensive assessment of cognitive function that evaluates 11 cognitive domains. To assess mood, we used the profile of mood states 2nd edition (POMS2), a comprehensive mood assessment tool that evaluates mood across seven categories, and the fatigue visual analog scale (VAS).

## 2 Materials and methods

### 2.1 Pharmacokinetic study

#### 2.1.1 Study design

We conducted an open-label, two-phase, two-dose sequential study that included a screening test (SCR) and two intake tests (VISIT1 and VISIT2). There was a two-week washout period between VISIT1 and VISIT2. We conducted this study at the Suntory World Research Center in Kyoto, Japan. Table 1 summarizes the schedule of the study. All subjects arrived at the center in a fasting state, having refrained from eating since 10:00 pm the previous night for all tests.

**Table 1:** Schedule of pharmacokinetic study^a^.

Parameters	Agreement	SCR	Test period		
			VISIT 1	Washout	VISIT 2
			First intake	2 weeks	Second intake
Mailing and Web	●	ー	ー	ー	ー
Visit	ー	●	●	ー	●
Medical interview	ー	●	●	ー	●
Vital sign	ー	●	●	ー	●
Blood test	ー	●	ー	ー	ー
Urine test	ー	●	ー	ー	ー
3-HB level	ー	ー	●	ー	●

^a● and – symbols refer to points that have been implemented and not been implemented, respectively.^

#### 2.1.2 Subjects

We recruited 16 subjects based on specific inclusion criteria: individuals who (1) completely understood the significance, contents, and objective of the study and provided written consent to participate, and (2) were healthy Japanese men aged between 40 and 60 years old. From this group, we selected 10 subjects who did not meet any of the following exclusion criteria: (1) having a diet restricted in carbohydrates within the previous 2 weeks, (2) regular consumption of MCT oil or other health foods related to ketones, (3) a 3-HB level above 0.076 mM (the upper limit of the reference value at the clinic) at SCR, and (4) regular engagement in strenuous physical activity.

#### 2.1.3 Intervention

The test food consisted of a 280 mL PET-bottled beverage containing 2 g of 3-HB for VISIT1 and 4 g for VISIT2. Table 2 presents the composition of the beverage. For the 3-HB content, we used a 34 wt.% aqueous solution of 3-HB provided by Osaka Gas Co., Ltd. The beverage preparation was under the supervision of the Suntory Global Innovation Center Ltd. Each subject consumed one bottle within 3 min on an empty stomach. Safety monitoring was performed throughout the study.

**Table 2:** Contents of beverages for pharmacokinetic study.

Parameters	2 g 3-HB	4 g 3-HB
Calories (kcal)	8	16
Proteins (g)	0	0
Lipids (g)	0	0
Carbohydrates (g)	0	0
Sodium (g)	0.001–0.003	0.001–0.003
3-HB (g)	2	4

#### 2.1.4 Serum 3-HB levels

For VISIT1 and VISIT2, serum 3-HB level measurement was based on 3HB-L Reagent KAINOS (KAINOS Laboratories, Inc., Tokyo, Japan) at specific intervals: 0, 5, 15, 30, 45, 60, and 120 min post-consumption of the test beverages.

#### 2.1.5 Construction of compartment models

**Figure 1:**
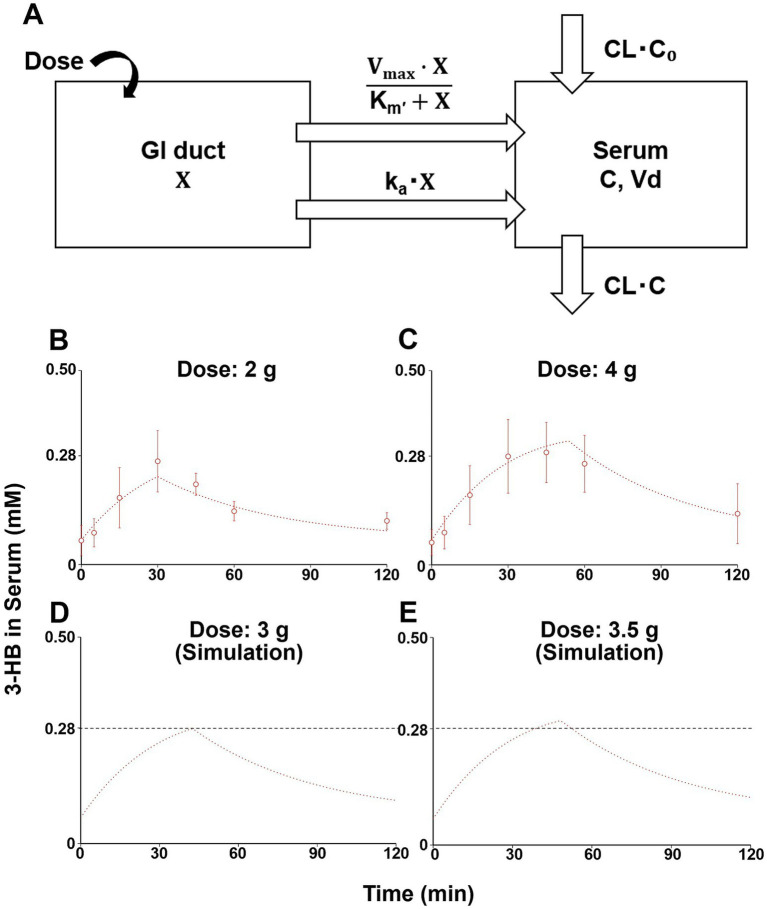
A one-compartment model describing serum 3-HB concentration profiles and simulation of serum 3-HB concentration. The one-compartment model includes saturable and non-saturable gastrointestinal absorption pathways, biosynthesis, and the linear elimination of 3-HB **(A)**. The one-compartment model was fitted to the average serum 3-HB concentration of 10 healthy subjects given 2 and 4 g of 3-HB (open circles), and straight lines represented fitted ones **(B,C)**. Serum 3-HB concentration profiles after intakes of 3 and 3.5 g of 3-HB were simulated using the one-compartment model, and the black dashed line indicates the threshold concentration (0.28 mM) **(D,E)**. Each value represents mean ± SD (*n* = 3–6). X, amount of 3-HB in GI duct compartment; C, serum 3-HB concentration, CL, systemic clearance; C_0_, estimated initial serum 3-HB concentration; k_a_, absorption rate constant; K_m_’, product of Michaelis constant and volume of GI duct compartment; Vd, volume of distribution; V_max_, maximum absorption rate; and CL•C_0_, endogenous production rate of 3-HB.

The previously established compartment model that described the pharmacokinetics of 3-HB after oral ingestion of (R)-3-hydroxybutyl (R)-3-hydroxybutyrate (3-HB monoester) (16) was first cited. This model considered two gut compartments, saturable and non-saturable elimination pathways, and negative feedback effect on endogenous production (16); whereas, we used a one-compartment model with one gastrointestinal (GI) duct compartment without considering feedback effect due to a limited sampling period after a single administration. In addition, our model includes both saturable and non-saturable gastrointestinal absorption pathways, in addition to biosynthesis and non-saturable clearance of 3-HB (Figure 1A). Based on this model, the following initial condition and mass-balance equations for the amount of 3-HB in GI duct compartment (X) and serum 3-HB concentration (C) were obtained:

At 0:


(1) X=Dose

(2) $$C=C_{0}$$

From 0:


(3) $$\frac{dX}{dt}=-\frac{V_{\text{max}}\times X}{K_{m\prime }+X}-k_{a}\times X$$

(4) $$V_{d}\times \frac{dC}{dt}=CL\times C_{0}+\frac{V_{\text{max}}\times X}{K_{m\prime }+X}+k_{a}\times X-CL\times C$$

where CL, C_0_, k_a_, K_m_’, Vd, and V_max_ represent systemic clearance, initial serum 3-HB concentration, absorption rate constant, product of Michaelis constant and volume of GI duct compartment, volume of distribution, and maximum absorption rate, respectively. The endogenous production rate of 3-HB was assumed to be unchanged, even after 3-HB ingestion, and therefore represented as CL•C_0_. The average serum 3-HB concentration profiles at 2 and 4 g of oral 3-HB ingestion were used as input data, and the six parameters were estimated by simultaneous non-linear least-squares fitting of the compartment model using the Napp nonlinear regression analysis program (version 2.3.1) (17).

### 2.2 Efficacy study

#### 2.2.1 Study design

We conducted a randomized, placebo-controlled, double-blind, crossover study that included SCR and two intake tests (VISIT1 and VISIT2). There was a four-week washout period between VISIT1 and VISIT2. We conducted this study at the Nihonbashi Sakura Clinic in Tokyo, Japan. Table 3 lists the study schedule. All subjects were required to arrive at the clinic in a fasting state, having refrained from eating since 10:00 p.m. the previous night for all tests.

**Table 3:** Schedule of efficacy study^a^.

Parameters	Agreement	SCR	Test period		
			VISIT 1	Washout	VISIT 2
			First intake	4 weeks	Second intake
Mailing and Web	●	ー	ー	ー	ー
Visit	ー	●	●	ー	●
Medical interview	ー	●	●	ー	●
Vital sign	ー	●	ー	ー	ー
Blood test	ー	●	ー	ー	ー
Urine test	ー	●	ー	ー	ー
POMS2^b^	ー	●	●	ー	●
VAS^c^	ー	●	●	ー	●
SAT^d^	ー	●	●	ー	●
Cognitrax	ー	ー	●	ー	●

^a● and – symbols refer to points that have been implemented and not been implemented, respectively.^

^bProfile of mood states.^

^cVisual analog scale.^

^dSerial arithmetic test.^

#### 2.2.2 Subjects

We recruited 68 subjects who met the following inclusion criteria: individuals who (1) completely understood the significance, content, and objective of the study and provided written consent to participate, and (2) were healthy Japanese men and women aged between 40 and 65 years old. From this group, we selected 40 subjects who did not meet any of the following exclusion criteria: (1) those who did not exhibit an increase in the fatigue–inertia score of the POMS2 following the SAT during SCR; (2) those who did not show an increase in the fatigue VAS score following the SAT in SCR; (3) regular consumers of MCT oil and other health foods related to ketones; and (4) regular users of medications, health foods, or supplements that could affect brain health.

#### 2.2.3 Randomization and blinding

Overall, 40 eligible subjects were randomized into two groups, with 20 subjects each allocated to Group I and Group II. The randomization process employed sorting blocks based on their age, sex, body mass index (BMI), and vigor–activity from the POMS2 as allocation factors. Group I consumed the active beverage on VISIT1 and the placebo one on VISIT2, whereas Group II consumed the placebo beverage on VISIT1 and the active one on VISIT2. The investigators and subjects were double-blinded to treatment regimens. A third-party allocation agency managed allocation information and ensured that double-blindness was maintained until the data were analyzed. The sample size was determined as the maximum feasible number for this exploratory study.

#### 2.2.4 Intervention

Both active and placebo foods were 190-mL PET bottled beverages. The active beverage contained 3.5 g of 3-HB, whereas the placebo did not. The two beverages could not be distinguished by appearance or smell. Table 4 lists the detailed contents of the beverages. The 40 wt% aqueous solution of 3-HB used for the active beverage was provided by Osaka Gas Co. (Osaka, Japan). The beverage preparation was under the supervision of Suntory Global Innovation Center Ltd. Each subject consumed one bottle within 3 min on an empty stomach. Safety monitoring was performed throughout the study.

**Table 4:** Contents of beverages for efficacy study.

Parameters	Active	Placebo
Calories (kcal)	14	0
Proteins (g)	0	0
Lipids (g)	0	0
Carbohydrates (g)	0	0
Sodium (g)	0–0.1	0–0.1
3-HB (g)	3.5	0

#### 2.2.5 Study outcomes

##### 2.2.5.1 Outline

**Figure 2:**
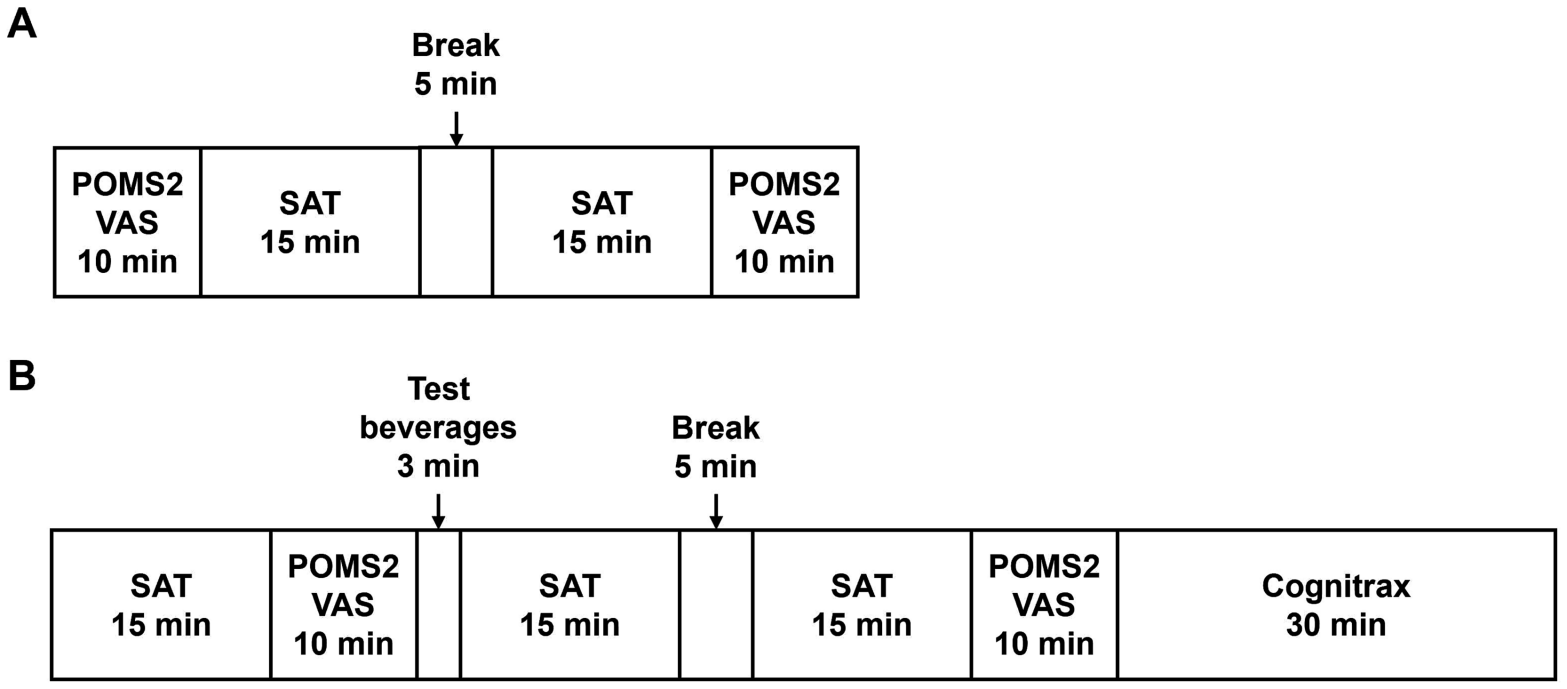
Schedule of efficacy study: SCR **(A)**, VISIT1 and VISIT2 **(B)**.

Figure 2 outlines the schedule of the SCR, VISIT1, and VISIT2. During the SCR, subjects were assessed by POMS2 and fatigue VAS before and after the SAT to assess whether the brain had expended energy and entered a fatigued state due to the SAT (Figure 2A). For VISIT1 and VISIT2, the SAT was performed for 15 min, followed by assessment using POMS2 and fatigue VAS (Figure 2B). After the subjects consumed the test beverage, the SAT was performed twice for 15 min each, with subsequent assessments using POMS2 and fatigue VAS. Finally, the Cognitrax (Health Solutions, Inc., Tokyo, Japan) was administered. The study endpoints for cognitive function included the changes in total responses and correct answers on the SAT, and each cognitive function domain of the Cognitrax. The mood endpoints included the changes in each item of the POMS2 and fatigue VAS.

##### 2.2.5.2 Cognitrax

The Cognitrax is a computerized cognitive functional test based on an assessment developed by CNS Vital Signs, Inc., North Carolina, USA (18). This online test comprises seven distinct tests for items, including verbal memory, visual memory, finger-tapping, symbol digit coding, stroop, attention shift, and sustained processing. The assessment evaluates multiple cognitive domains, including neurocognitive index, composite memory, verbal memory, visual memory, psychomotor speed, reaction time, complex attention, cognitive flexibility, processing speed, executive function, simple attention, and motor speed. Scores were normalized by comparing them with those of similar age groups.

##### 2.2.5.3 Serial arithmetic test (SAT)

The SAT was performed using the Uchida–Kraepelin test forms. The Uchida–Kraepelin test is a simple one-digit addition test that can be used as a fatigue stressor and evaluate attentional function (19). During the SCR, subjects undertook two 15-min addition tasks (Figure 2A). In VISIT1 and VISIT2, subjects performed one 15-min addition task before consuming the test beverage and two subsequent 15-min addition tasks post-ingestion (Figure 2B). In the case of two consecutive 15-min tasks, there was a 5-min break in between.

##### 2.2.5.4 POMS2 and fatigue VAS

The POMS2 provided by Success Bell K.K. (Hiroshima, Japan) is a comprehensive mood assessment tool (20). It involves subjects responding to 65 questions on a 5-point scale ranging from “not at all” to “extremely.” This test evaluates mood across seven categories, including anger–hostility, confusion–bewilderment, depression–dejection, fatigue–inertia, tension–anxiety, vigor-activity, and friendliness. The test generates the total mood disturbance (TMD) score, which represents the overall negative mood state. Subjects reported how they felt “now,” thereby assessing their temporary mood.

Furthermore, fatigue levels were measured using a VAS. This scale prompted subjects to mark their current level of tiredness, ranging from “the best feeling of not feeling tired at all” to “the worst feeling of being so exhausted that you cannot do anything.”

#### 2.2.6 Statistical analysis

For baseline characteristics, differences in each parameter at the time of SCR were statistically determined using an independent two-sample t-test, except the sex ratio, which is based on Fisher’s exact test. For the study endpoints, treatment effects were evaluated from a linear mixed model that included sequence, period, and treatment as fixed effects, with subjects as a random effect. The significance threshold was set at 5% (two-sided). Data management and statistical analyses were performed using multiple platforms, including IBM SPSS Statistics 25 or higher (International Business Machines Corporation, NC, USA), R version 3.5.2 (R Foundation for Statistical Computing, Vienna, Austria), Microsoft Excel 2013 (Microsoft Corporation, VA, USA), and JMP version 14.0.0 (SAS Institute Inc., Cary, NC, USA).

## 3 Results

### 3.1 Pharmacokinetic study

#### 3.1.1 Subject flow and baseline characteristics

**Figure 3:**
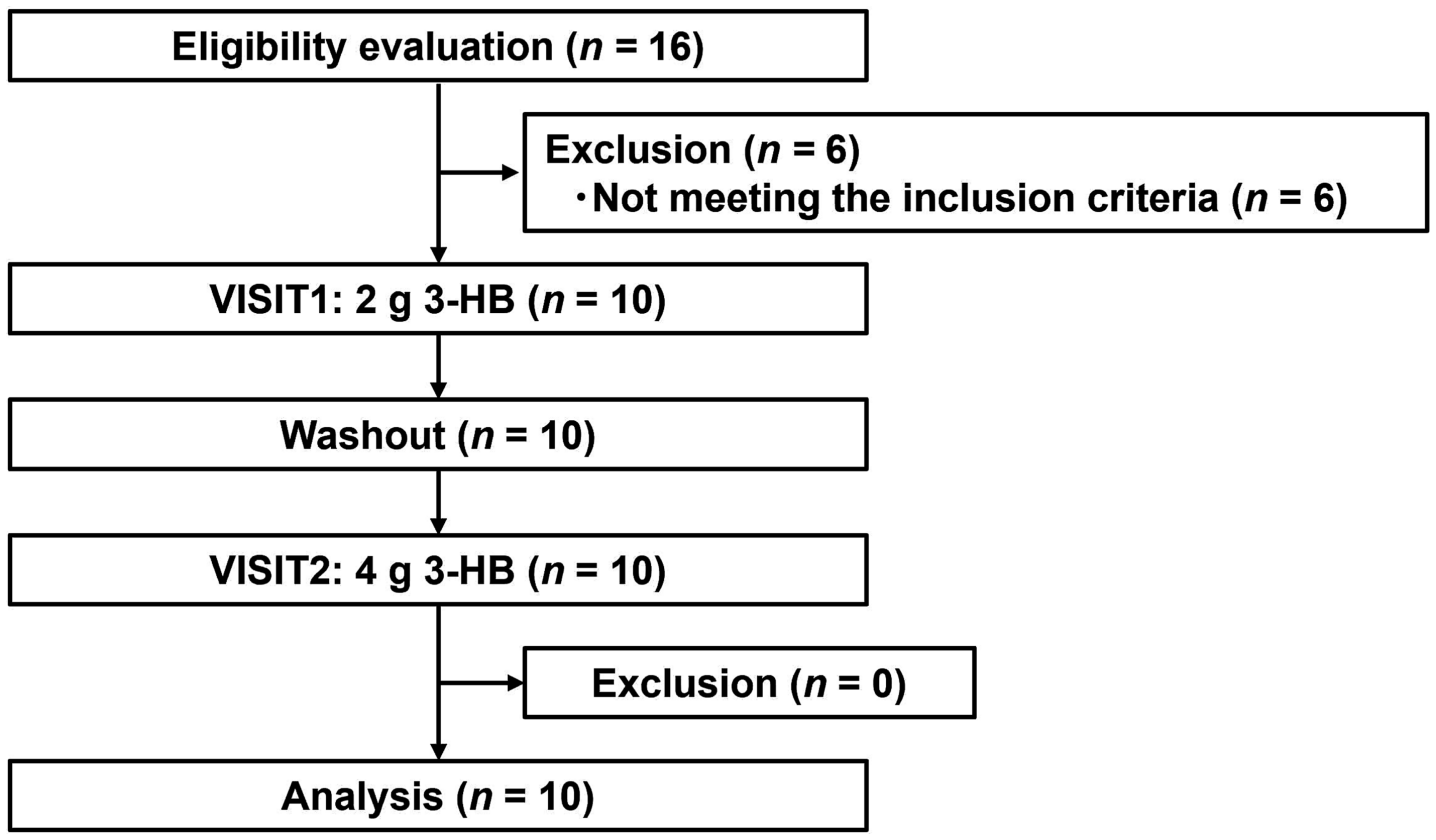
Flowchart of pharmacokinetic study.

Figure 3 depicts the flowchart of the pharmacokinetic study subjects. From the initial 16 individual subjects who consented to participate, we selected 10 eligible subjects. All 10 subjects participated in both VISIT1 and VISIT2. Table 5 summarizes the backgrounds of the subjects. The average age was 49.1 ± 6.1 years old. There was no adverse event directly linked to the consumption of the test beverages.

**Table 5:** Baseline characteristics of pharmacokinetic study.

Parameters^a^	Total (*n* = 10)
Age (years)	49.1 ± 5.8
Sex (male/female)	10/10
Height (cm)	171.7 ± 6.6
Weight (kg)	67.8 ± 8.0
BMI^b^ (kg m^−2^)	22.9 ± 1.9
SBP^c^ (mmHg)	128.3 ± 8.8
DBP^d^ (mmHg)	81.7 ± 7.5
PR^e^(/min)	68.0 ± 6.0
Intake rate (%)	100 ± 0.0

^aMean ± SD.^

^bBody mass index.^

^cSystolic blood pressure.^

^dDiastolic blood pressure.^

^ePulse rate.^

#### 3.1.2 Serum 3-HB levels

Figures 1B,C depict the changes in serum 3-HB levels following the ingestion of the test beverages. For the group that ingested 2 g of 3-HB, the initial serum concentration was 0.062 ± 0.039 mM, which increased to a maximum of 0.266 ± 0.079 mM 30 min after ingestion. Moreover, the group that consumed 4 g of 3-HB started with a serum concentration of 0.057 ± 0.033 mM, achieving a maximum of 0.289 ± 0.077 mM 45 min after consumption. These results suggest that the optimal dose of 3-HB to achieve a C_max_ exceeding 0.28 mM lies between 2 and 4 g.

#### 3.1.3 Construction of a compartmental model

The simultaneous fitting of the compartment model (Figure 1A) to the serum 3-HB concentration profiles at 2 and 4 g of oral 3-HB ingestion yielded fitted lines close to the observed values (Figures 1B,C). They yielded the six parameters estimated (Supplementary Table S1). The calculated SD value of K_m_’ was close to the estimated value, thus suggesting uncertainty for this parameter; however, those of the other five parameters were lower than the estimated values (Supplementary Table S1). The fitting was also performed on the compartment model, including only the non-saturable (Supplementary Figure S1A) and saturable (Supplementary Figure S1D) absorption pathway alone. The fitted line of the former model cannot fully explain the serum 3-HB concentration profile, especially at a higher dose (Supplementary Figures S1B,C), whereas that of the latter model was close to the observed values (Supplementary Figures S1E,F) and yielded comparable Akaike’s information criterion (AIC), but higher final sum of square values compared with the final model shown in Figure 1A (Supplementary Table S1). Therefore, we adopted the final model and conducted simulations (Figures 1D,E) to estimate the minimum dose necessary to reach the target serum 3-HB concentration (0.28 mM). Through these simulations, the minimum dose required to achieve the target 0.28-mM serum 3-HB was estimated to be 3.5 g (Figures 1D,E). This dose (3.5 g) was utilized in the subsequent clinical study to evaluate the effects of 3-HB on cognitive function and mood.

### 3.2 Efficacy study

#### 3.2.1 Subject flow and baseline characteristics

**Figure 4:**
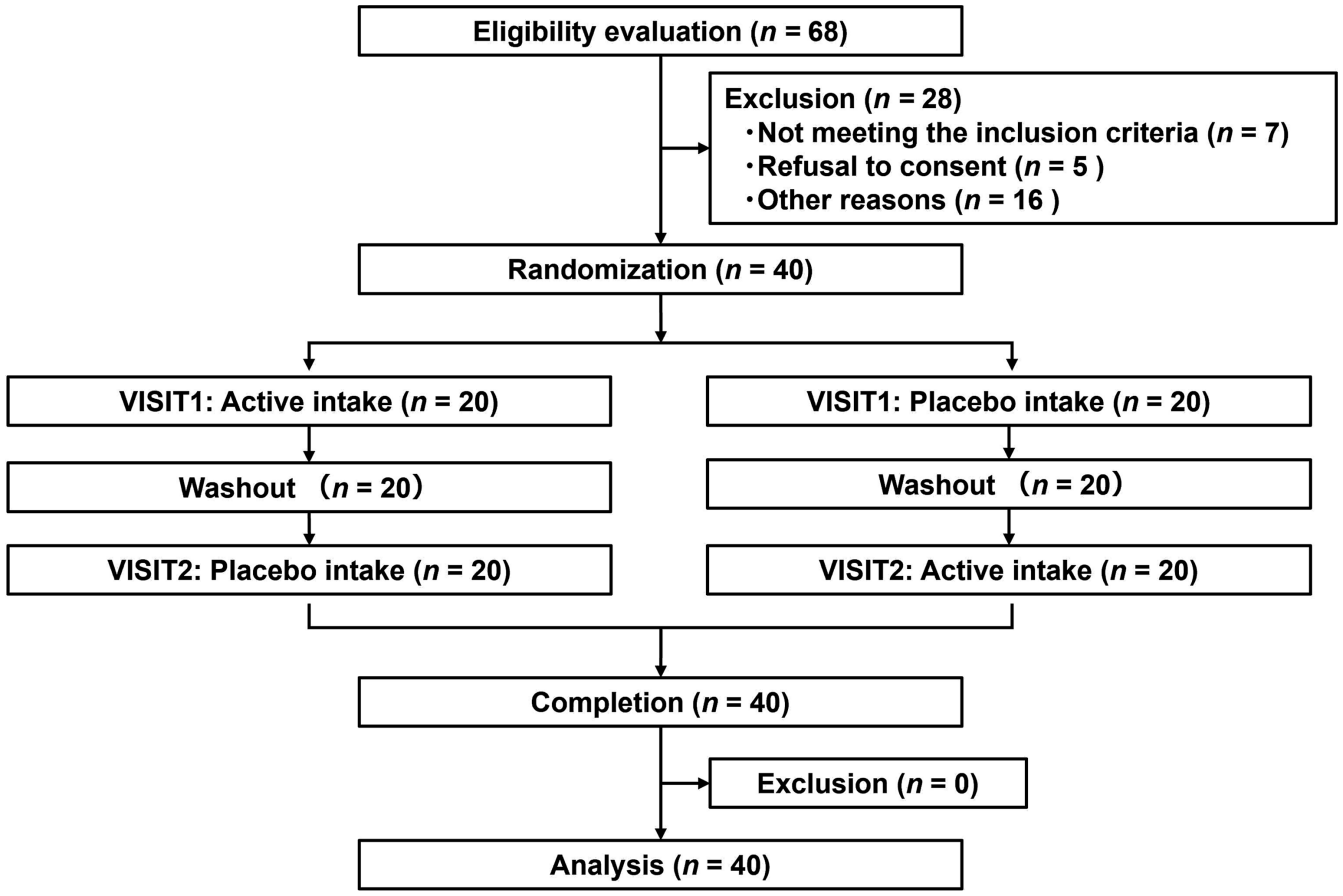
Flowchart of efficacy study.

Figure 4 depicts the flowchart of the efficacy study subjects. We selected 40 eligible subjects from 68 and divided them into two groups (Groups I and II), each including 20 subjects. All 40 subjects completed the study. Table 6 summarizes the background of the subjects, indicating an average age of 52.1 ± 6.4 years old. The assignment factors, age, gender, BMI, and vigor-activity of POMS2 were not significantly different between groups. All subjects consumed their assigned test beverages. Moreover, selected subjects exhibited increased fatigue following the SAT, as evidenced by the measures of fatigue–inertia in the POMS2 and the fatigue VAS in SCR (Table 7). There was no adverse event directly linked to the consumption of the test beverages.

**Table 6:** Baseline characteristics of efficacy study.

Parameters^a^	Total (*n* = 40)	Group I (*n* = 20)	Group II (*n* = 20)	*p* value^b^
Age (years)	52.1 ± 6.4	52.0 ± 6.4	52.2 ± 6.5	0.922
Sex (male/female)	16/24	7/13	9/11	0.748
Height(cm)	164.4 ± 8.7	163.5 ± 8.8	165.3 ± 8.7	0.528
Weight(kg)	57.4 ± 9.1	56.7 ± 7.6	58.0 ± 10.6	0.665
BMI^c^ (kg m^−2^)	21.2 ± 2.2	21.2 ± 2.0	21.1 ± 2.6	0.940
SBP^d^ (mmHg)	111.4 ± 12.2	110.9 ± 10.7	111.9 ± 13.7	0.789
DBP^e^ (mmHg)	72.9 ± 9.9	72.6 ± 8.4	73.2 ± 11.3	0.838
PR^f^ (/min)	65.8 ± 10.0	68.0 ± 11.9	63.7 ± 7.3	0.176
Vigor-activity of POMS2	9.53 ± 7.01	9.55 ± 7.99	9.50 ± 6.09	0.982
Intake rate (%)	100 ± 0.0	100 ± 0.0	100 ± 0.0	1.000

^aMean ± SD.^

^bFisher’s t-test was performed for sex, and other p values are from independent two-sample t-tests.^

^cBody mass index.^

^dSystolic blood pressure.^

^eDiastolic blood pressure.^

^fPulse rate.^

**Table 7:** Fatigue before and after SAT in SCR.

Parameters	Before SAT^a^ (*n* = 40)	After SAT^a^ (*n* = 40)	*p* value^b^
Fatigue-inertia of POMS2	6.5 ± 5.5	16.1 ± 5.4	* < 0.001
Fatigue VAS	40.1 ± 21.2	73.8 ± 15.4	* < 0.001

^aMean ± SD.^

^b^*p* value is from corresponding t-test, *p < 0.05.

#### 3.2.2 Effects on cognitive function

Regarding the SAT, changes in the total responses and correct answers for the first and second 15-min sessions in the active groups were significantly higher than in the placebo groups (Table 8). Raw data are shown in Supplementary Table S2. As for the Cognitrax, no significant differences were observed between the active and placebo groups in the neurocognitive index, which indicates overall cognitive function, nor across all 11 assessed cognitive domains (Table 9). No significant effects of 3-HB were observed even on the scores of the seven tests before they were converted to the cognitive domains (data not shown).

**Table 8:** SAT assessment.

Parameters	Active^a^ (*n* = 40)	Placebo^a^ (*n* = 40)	Active-Placebo^b^ (*n* = 40)	*p* value^c^
First half				
Δ Total responses	82.5 ± 34.7	53.7 ± 51.2	28.8 (12.1 to 45.5)	* < 0.001
Δ Correct answers	80.4 ± 34.2	53.1 ± 50.6	27.2 (10.2 to 44.2)	* < 0.001
Second half				
Δ Total responses	80.9 ± 52.3	41.8 ± 92.3	39.2 (11.4 to 66.9)	*0.007
Δ Correct answers	78.8 ± 50.8	41.5 ± 93.7	37.3 (9.0 to 65.6)	*0.011

^aMean ± SD.^

^b^Least square mean (95% CI).

^c^*p* value is from linear mixed model, *p < 0.05.

**Table 9:** Cognitrax assessment.

Parameters	Active^a^ (*n* = 40)	Placebo^a^ (*n* = 40)	Active-Placebo^b^ (*n* = 40)	*p* value^c^
NCI^d^	106.1 ± 8.5	106.6 ± 8.6	−0.6 (−2.0 to 0.8)	0.413
Composite memory	106.2 ± 15.9	108.8 ± 15.3	−2.6 (−5.7 to 0.6)	0.105
Verbal memory	108.2 ± 14.3	108.9 ± 15.0	−0.7 (−4.7 to 3.3)	0.726
Visual memory	102.2 ± 16.4	105.5 ± 16.6	−3.3 (−7.6 to 0.9)	0.120
Psychomotor speed	117.1 ± 14.3	116.2 ± 16.4	0.9 (−1.8 to 3.6)	0.500
Reaction time	97.5 ± 9.4	97.1 ± 9.6	0.4 (−2.3 to 3.0)	0.777
Complex attention	106.9 ± 11.0	107.6 ± 7.5	−0.7 (−3.6 to 2.2)	0.631
Cognitive flexibility	102.8 ± 11.7	103.5 ± 10.7	−0.7 (−3.0 to 1.6)	0.532
Processing speed	118.1 ± 14.1	119.0 ± 14.7	−0.9 (−3.2 to 1.4)	0.444
Executive function	103.4 ± 11.7	103.6 ± 11.0	−0.2 (−2.4 to 2.1)	0.893
Simple attention	101.1 ± 14.4	101.8 ± 11.8	−0.7 (−6.0 to 4.6)	0.804
Motor speed	110.9 ± 16.3	109.1 ± 18.0	1.8 (−1.1 to 4.7)	0.206

^aMean ± SD.^

^b^Least square mean (95% CI).

^c^*p* value is from linear mixed model, *p < 0.05.

^dNeurocognitive score index.^

#### 3.2.3 Effects on moods

In the POMS2, the changes in confusion-bewilderment, fatigue–inertia, and TMD scales in the placebo group were significantly higher than in the active group (Table 10). The changes in vigor–activity in the active group were higher than in the placebo group (Table 10). No other items exhibited significant differences (Table 10). Raw data are shown in Supplementary Table S3. The change in the fatigue VAS score in the placebo group was significantly higher than in the active group (Table 11). Raw data are shown in Supplementary Table S4.

**Table 10:** POMS2 assessment.

Parameters	Active^a^ (*n* = 40)	Placebo^a^ (*n* = 40)	Active-Placebo^b^ (*n* = 40)	*p* value^c^
Δ Anger-hostility	−1.0 ± 1.9	−0.5 ± 2.0	−0.5 (−1.3 to 0.2)	0.161
Δ Confusion-bewilderment	0.3 ± 2.6	1.4 ± 4.3	−1.2 (−2.3 to −0.0)	*0.043
Δ Depression-dejection	−0.3 ± 2.4	−0.7 ± 2.4	0.35 (−0.3 to 1.0)	0.287
Δ Fatigue-inertia	1.5 ± 2.4	3.0 ± 3.9	−1.6 (−2.6 to −0.5)	*0.005
Δ Tension-anxiety	0.1 ± 3.3	0.3 ± 3.9	−0.2 (−1.2 to 0.8)	0.674
*Δ* Vigor-activity	−0.1 ± 3.5	−1.7 ± 3.3	1.6 (0.4 to 2.9)	*0.011
Δ Friendliness	−0.5 ± 1.6	−1.2 ± 2.5	0.7 (0.2 to 1.5)	0.123
Δ TMD	0.6 ± 11.6	5.3 ± 14.6	−4.7 (−8.5 to −0.9)	*0.017

^aMean ± SD.^

^b^Least square mean (95% CI).

^c^*p* value is from linear mixed model, *p < 0.05.

**Table 11:** Fatigue VAS assessment.

Active^a^ (*n* = 40)	Placebo^a^ (*n* = 40)	Active-Placebo^b^ (*n* = 40)	*p* value^c^
7.2 ± 14.1	13.8 ± 17.4	−6.6 (−11.9 to −1.4)	*0.015

^aMean ± SD.^

^b^Least square mean (95% CI).

^c^*p* value is from linear mixed model, *p < 0.05.

## 4 Discussion

In this research, we developed a one-compartment model for the oral intake of 3-HB to estimate its optimal dose (3.5 g) in the pharmacokinetic study and demonstrated partial efficacy on cognitive function and broad efficacy on mood with a single oral dose of 3.5 g of 3-HB in healthy subjects in the efficacy study. With respect to the available literature, this is the first study to demonstrate the benefits of ingesting 3-HB on indicators of brain health.

In the pharmacokinetic study, we developed a one-compartment model (Figure 1A) based on 3-HB level data after consuming beverages containing 2 and 4 g of 3-HB. Accordingly, we estimated that 3.5 g of 3-HB was the minimum dose required to achieve a serum concentration of 0.28 mM for inducing ketone metabolism in the brain (Figures 1D, E) (14). This model incorporated a saturable gastrointestinal absorption pathway (Figure 1A), which may be compatible with that 3-HB is a substrate of proton-coupled and sodium-coupled monocarboxylate transporters, which are expressed in the small intestine (21, 22). To simplify the model, we assumed that the elimination rate of 3-HB could be the same as the synthesis rate, as plasma 3-HB levels are typically maintained at a steady state under normal conditions without intervention (23). Regarding the validity of the obtained parameter values (Supplementary Table S1), the CL value estimated in this study was approximately 1.82 L/min (109 L/h, Supplementary Table S1), which was closely aligned with the previously reported values of around 96.7 and 112 L/h (16). The basal concentration (C_0_) was estimated at approximately 0.063 mM (Supplementary Table S1), which almost coincided with the reported value of approximately 0.070 mM (16), implying the validity of these values. However, the estimated Vd value was 85.7 L (Supplementary Table S1), which was larger than the reported value of 12 L (16). This discrepancy might have stemmed from differences in the administered compounds (3-HB vs. 3-HB ester) or variations in 3-HB levels in plasma or serum between the studies: the reported mean C_max_ values were approximately higher than those estimated in this study by factors of 10–20, which may be due to saturation in the tissue distribution process in the previous studies. The proposed model was established by fitting the data from a limited number of male-only study subjects and sampling period, so perfect generalization may be difficult. Therefore, it may be necessary to validate the model in other populations with different ages and nationalities, and if this does not work, we need to consider building a more sophisticated and generalizable model, which could include feedback effect on endogenous production of 3-HB.

In the efficacy study, we evaluated the effects of orally ingesting 3.5 g of 3-HB on cognitive function and mood. Regarding cognitive function, 3-HB significantly increased total responses and correct answers in the SAT using the Uchida–Kraepelin test forms (Table 8). The SAT involves continuously repeating a simple task and is used to test attention among cognitive functions (24–26). Thus, the intake of 3.5 g of 3-HB may arguably improve attentional function. Baba Y et al. evaluated the effects of a single intake of 2070 mg matcha or 66.6 mg caffeine on middle-aged healthy subjects in the SAT using the Uchida–Kraepelin test forms, and identified a significant difference in the rate of change in total responses from baseline between the matcha or caffeine groups and the placebo group, ranging from approximately +3% to +8% (25). In our present study, the difference in the rate of change in total responses between the active and the placebo groups was +3.58% (first half) and + 4.80% (second half) (calculated from Supplementary Table S2). This equivalency of the values may suggest that the effects of 3.5 g of 3-HB on attentional function observed in our study were psychologically meaningful. Despite the positive effects observed in the SAT, there was no significant effect on any cognitive domains assessed by the Cognitrax (Table 9). To consider the reasons for this discrepancy, we focused on previous studies that implemented both the SAT using the Uchida–Kraepelin test forms and Cognitrax (25, 27). In the above-mentioned study by Baba Y et al., they found that the caffeine group not only had positive effects on attentional function, as assessed by the SAT, but also demonstrated a significantly faster reaction time providing correct answers in the attention shift test in the Cognitrax compared to the placebo group, regarding the amount of change from baseline (25). Conversely, Kanatome A et al. evaluated the effects of a single intake of 35 mg mature hop bitter acids on healthy adults by both tests and did not confirm any significant effects in the SAT, while they found that, in the Cognitrax after ingestion, the number of correct responses in the attention shift test and the executive function score were significantly higher in the active group than in the placebo group (27). An overview of the results of the study of Baba Y et al., Kanatome A et al., and our present study suggests that the relevance between the results of SAT and Cognitrax may vary depending on factors such as study subjects, study designs, and test food profiles. Although the limited effects of 3-HB on attentional function observed in the present study, detected only in the SAT, should be interpreted cautiously in its generalization, it can be suggested that a dosage of 3.5 g of 3-HB may have positive effects on cognitive function measured by the Cognitrax, depending on the conditions.

Regarding mood, 3-HB significantly suppressed scores for confusion–bewilderment, fatigue–inertia, and TMD scales, while enhanced vigor-activity in the POMS2 (Table 10). Confusion–bewilderment is characterized by bewilderment and cognitive inefficiency, and fatigue–inertia represents a mood of weariness, inertia, and low energy, while vigor-activity is defined by adjectives painting a mood of vigorousness, ebullience, and high energy. The TMD scales are obtained by subtracting the vigor-activity score from the sum total of five scores, anger-hostility, confusion-bewilderment, depression-dejection, fatigue-inertia, and tension-anxiety, and is used as a global indication of mood disturbance, emotional or psychological distress, or subjective well-being. Significant improvements of those indicators with 3.5 g of 3-HB intake may indicate the positive effects of this dose of 3-HB on a wide range of mood state. Setoguchi Y et al. evaluated the effects of a single intake of glucose-containing tablet candy on healthy adults in the POMS2 and identified a significant difference in the changes of the scores of vigor–activity and TMD from baseline between the candy and placebo groups, with differences of +4.6 and − 2.4 points, respectively (28). In our present study, the differences in the changes in the scores of vigor–activity and TMD between active and placebo groups were + 1.6 and − 4.7 points, respectively (Table 10). This similarity of the values may suggest that the effects of 3.5 g of 3-HB on moods found in our study were psychologically meaningful. In addition, we demonstrated that 3-HB suppressed the increase in fatigue VAS scores. This result may substantiate the positive effects of 3-HB on moods shown in the POMS2, particularly in fatigue-inertia.

One of the limitations of the efficacy study is that it was unclear whether orally administered 3-HB was utilized as the brain energy. In addition to serving as an energy substrate, 3-HB may have other effects on the brain, such as reducing oxidative stress by inhibiting histone deacetylases HDACs (29). To better understand the mechanisms of action of 3-HB in the brain, further detailed studies using neuroimaging and biochemical markers are required. Another limitation is the specific conditions of the study, in which the subjects were required to come to the clinic in a fasting state to accurately assess the effects of 3-HB as an energy substrate with high sensitivity. Therefore, it is unclear whether the present findings can be generalized to the case of 3-HB intake when people are full or during a meal. Future research should rigorously examine the dietary conditions under which 3-HB intake exerts positive effects on brain health. A further limitation is that we were unable to measure serum concentrations of 3-HB in the efficacy study due to difficulties in performing blood sampling during the assessments of cognitive function and mood, implying that the validity of the target C_max_ of 0.28 mM might not be fully confirmed. Recently, a technology has been developed to monitor 3HB levels in the body over time by simply attaching it to the upper arm (30),which may allow measurement of the levels of 3-HB during neuropsychological or psychometric assessments. Finally, the sample size for this exploratory study was determined as the maximum feasible number of 40 participants; however, the number may be insufficient, especially considering the multiple comparisons across different cognitive and mood measures. As this limitation may raise the risk of Type I errors (false positives), it would be beneficial to conduct confirmatory trials with larger sample sizes to more accurately validate the action of 3- HB in the brain.

## 5 Conclusion

We successfully developed a pharmacokinetic model for oral administration of 3-HB. Moreover, a single dose of 3.5 g of 3-HB optimized by the model, positively affected attentional function, as assessed by the SAT, and improved various mood parameters measured by POMS2 and fatigue VAS in healthy adults, although no positive effects were confirmed for cognitive domains as assessed by the Cognitrax.

## 6 Study approval

The pharmacokinetic and efficacy studies were conducted in accordance with the Ethical Guidelines for Medical Research Involving Human Subjects (partially revised in 2017 by the Ministry of Education, Culture, Sports, Science, and Technology and the Ministry of Health, Labor, and Welfare) and complied with the tenets of the Declaration of Helsinki (revised in October 2013 by the World Medical Association Fortaleza General Assembly). The pharmacokinetic study was reviewed and approved by the Bioethics Committee of Miura Clinic (IRB number: 17000161). The efficacy study was reviewed and approved by the Ethics Committee of the Yoga Allergy Clinic (IRB number: 21000023). Summaries of both studies were registered in the University Hospital Medical Information Network Clinical Trial Registration System (UMIN-CTR) (UMIN000042095, UMIN000046666).

## Data Availability

The original contributions presented in the study are included in the article/Supplementary material, further inquiries can be directed to the corresponding author.

## Supplementary material

SUPPLEMENTARY FIGURE S1 One-compartment models describing serum 3-HB concentration profiles and simulation of serum 3-HB concentration. One model includes non-saturable gastrointestinal absorption pathways, biosynthesis, and the linear elimination of 3-HB **(A)**. The other model includes saturable gastrointestinal absorption pathway, biosynthesis, and the linear elimination of 3-HB **(D)**. The former model was fitted to the average serum 3-HB concentration of 10 healthy subjects given 2 and 4 g of 3-HB (open circles), and straight lines represented fitted ones **(B,C)**. Similar fitting was performed for the latter model **(E,F)**. Each value represents mean ± SD (*n* = 3–6). X, amount of 3-HB in GI duct compartment; C, serum 3-HB concentration; CL, systemic clearance; C0, estimated initial serum 3-HB concentration; ka, absorption rate constant; Km', product of Michaelis constant and volume of GI duct compartment; Vd, volume of distribution; Vmax, maximum absorption rate; and CL•C0, endogenous production rate of 3-HB.
