# Supplementary figures and images for: Optimizing oral 3-hydroxybutyrate dosage using pharmacokinetic model to improve cognitive function and mood in healthy subjects
Source: Front Nutr. 2025 Jan 8;11:1470331. doi: 10.3389/fnut.2024.1470331 (PMC11758625; doi:10.3389/fnut.2024.1470331)

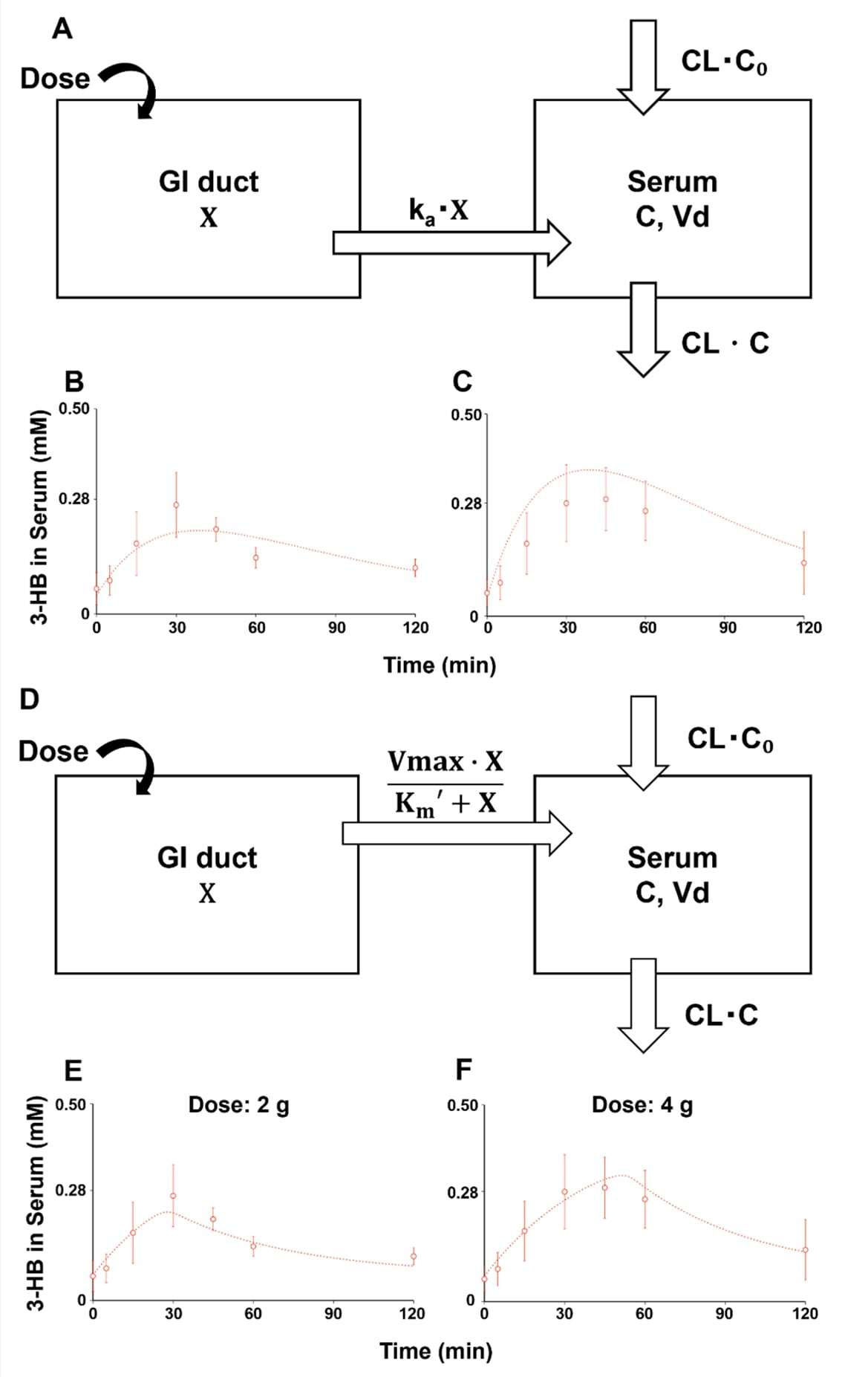

Supplement: SUPPLEMENTARY FIGURE S1 — One-compartment models describing serum 3-HB concentration profiles and simulation of serum 3-HB concentration. One model includes non-saturable gastrointestinal absorption pathways, biosynthesis, and the linear elimination of 3-HB (A). The other model includes saturable gastrointestinal absorption pathway, biosynthesis, and the linear elimination of 3-HB (D). The former model was fitted to the average serum 3-HB concentration of 10 healthy subjects given 2 and 4 g of 3-HB (open circles), and straight lines represented fitted ones (B,C). Similar fitting was performed for the latter model (E,F). Each value represents mean ± SD (n = 3–6). X, amount of 3-HB in GI duct compartment; C, serum 3-HB concentration; CL, systemic clearance; C0, estimated initial serum 3-HB concentration; ka, absorption rate constant; Km', product of Michaelis constant and volume of GI duct compartment; Vd, volume of distribution; Vmax, maximum absorption rate; and CL•C0, endogenous production rate of 3-HB. [file Image_1.jpeg]
