# Supplementary material for: Optimizing oral 3-hydroxybutyrate dosage using pharmacokinetic model to improve cognitive function and mood in healthy subjects
Source: Front Nutr. 2025 Jan 8;11:1470331. doi: 10.3389/fnut.2024.1470331 (PMC11758625; doi:10.3389/fnut.2024.1470331)
Supplement: Supplementary file 2 [file Table_1.pdf]

Supplementary tables

Table S1. Parameters estimated by the fitting [a] and predictive accuracy of each model

Parameters or accuracy indices	Three types of models with different GI absorption processes: Saturable and non-saturable pathways (Figure 3A)	Non-saturable pathway alone (Figure S1A)	Saturable pathway alone (Figure S1D)
K_m' [b] (mmol)	0.000999 ± 0.000994	- [j]	0.813 ± 4.15
V_{max} [c] (mmol/min)	0.562 ± 0.151	- [j]	0.775 ± 0.185
Vd [d] (L)	85.7 ± 23.0	44.6 ± 134000	85.7 ± 13.8
CL [e] (L/min)	1.82 ± 0.42	1.15 ± 0.35	1.87 ± 0.32
k_a [f] (min^{-1})	0.00855 ± 0.00633	0.0258 ± 77.2	- [j]
C_0 [g] (mM)	0.0628 ± 0.0130	0.0453 ± 0.0190	0.0652 ± 0.0124
Final SS [h]	0.00561	0.0104	0.00643
AIC [i]	-60.6	-55.9	-60.7

a) Each value represents per human value (values ± calculated SD)
b) Product of Michaelis constant and volume of GI duct compartment
c) Maximum absorption rate
d) Volume of distribution
e) Systemic clearance
f) Absorption rate constant
g) Estimated initial serum 3-HB concentration
h) Final sum of square
i) Akaike’s information criterion
j) Not applicable

Table S2. SAT assessment

Parameters	Group	Before intake a)	After intake a)	
			First half	Second half
Total responses	Placebo	841.9 ± 216.2	895.6 ± 227.1	883.7 ± 222.3
	Active	828.4 ± 214.0	910.9 ± 229.1	909.3 ± 229.6
Correct answers	Placebo	834.3 ± 216.9	887.4 ± 226.8	875.8 ± 222.9
	Active	820.7 ± 214.2	901.1 ± 227.9	899.5 ± 229.1

a) Mean ± SD

Table S3. POMS2 assessment

Parameters	Group	Before intake a)	After intake a)
Anger−hostility	Placebo	5.4 ± 6.5	4.9 ± 6.6
	Active	6.2 ± 7.7	5.2 ± 7.1
Confusion−bewilderment	Placebo	12.6 ± 6.1	14.0 ± 6.8
	Active	12.8 ± 6.4	13.1 ± 6.3
Depression−dejection	Placebo	7.9 ± 9.1	7.2 ± 9.0
	Active	8.1 ± 9.3	7.7 ± 9.3
Fatigue−inertia	Placebo	7.4 ± 5.0	10.4 ± 6.0
	Active	7.7 ± 5.1	9.2 ± 5.8
Tension−anxiety	Placebo	13.8 ± 7.3	14.1 ± 6.4
	Active	13.9 ± 7.6	14.0 ± 7.2
Vigor−activity	Placebo	13.8 ± 6.7	12.1 ± 6.9
	Active	13.8 ± 7.0	13.7 ± 7.5
Friendliness	Placebo	11.7 ± 4.6	10.6 ± 5.0
	Active	11.8 ± 4.5	11.3 ± 4.8
TMD	Placebo	33.3 ± 32.1	38.6 ± 32.8
	Active	34.9 ± 33.5	35.5 ± 33.7

a) Mean ± SD

Table S4. Fatigue VAS assessment

Parameters	Before intake [a)]	After intake [a)]
Placebo	46.4 ± 19.4	60.2 ± 17.6
Active	46.4 ± 21.2	53.5 ± 19.6

a) Mean ± SD
